# The benefits and boundaries of psychological safety in simulation-based education: an integrative review

**DOI:** 10.1186/s12912-025-03575-y

**Published:** 2025-07-15

**Authors:** Anine Madsgaard, Alette Svellingen

**Affiliations:** VID Specialized University, Bergen, Norway

**Keywords:** Simulation-based learning, Experiential learning, Active learning, Teaching

## Abstract

**Background:**

Psychological safety has been highlighted as essential for student learning in simulation-based education (SBE). The pedagogical literature emphasizes that such learning situations must challenge learners and make them experience that something is at stake. Therefore, this study aimed to analyse how psychological safety in SBE is assured in recent research.

**Methods:**

The study employed an integrative review method. Databases were systematically searched for articles, and 2071 records were screened, of which 13 studies met the inclusion criteria.

**Findings:**

The findings of the study resulted in two themes related to psychological safety in SBE. Theme 1 demonstrates that psychological safety is warranted because simulation is an unsafe learning environment, while theme 2 illustrates that psychological safety is implemented to optimise students’ learning during SBE.

**Conclusion:**

This study reveals that establishing psychological safety in SBE is crucial due to the potential for students to feel anxious and stressed. Psychological safety in SBE is more nuanced than in traditional work environments in which the concept originated and is not merely about eliminating all discomfort during learning situations. Additionally, psychological safety is necessary for a challenging learning environment in which students feel safe enough to risk failure and be exposed to dilemmas, thereby facilitating learning opportunities.

**Clinical trial number:**

Not applicable.

## Introduction

Establishing a psychologically safe environment in simulation-based education (SBE) has garnered attention in recent years [1]. Participation in simulation can evoke feelings of exposure because students may fear revealing knowledge gaps and perceive feedback on performance as threatening [2, 3]. In this context, psychological safety becomes crucial. It is defined as a condition that allows individuals to feel safe and comfortable expressing themselves and to take interpersonal risks without fear of negative consequences [4, 5]. This concept is particularly important in high-stakes and uncertain interactive contexts in which anxiety can undermine learning outcomes. 

A psychologically safe environment fosters a culture in which discussing mistakes is both encouraged and accepted. This culture not only promotes comprehensive learning conditions, but also enables participants to expand their understanding and knowledge [6, 7]. In such an environment, participants feel respected, and ambitious goals are set and can be collaboratively pursued. Conversely, an environment characterized by high demands and expectations combined with low psychological safety can lead to fear, negative consequences and reprimands for those who choose to speak up.

Learning during SBE typically occurs within a socio-cultural context with a focus on social interaction and community participation [8]. This foundational perspective posits that knowledge is constructed thorough interpersonal interactions within specific contexts, and simulation aligns well with this view by fostering group interactions in a reality-based environment. Because of interactions students may experience exposure and vulnerability when their knowledge, skills and abilities are publicly visible to peers and facilitators [9]. In a complex learning environment such as SBE, it becomes crucial to intentionally create and sustain a sense of psychological safety.

However, pedagogical theory argues that overly easy, comfortable and safe educational environments create unfavourable learning situations [10]. The literature emphasizes the necessity of challenges for meaningful learning [11, 12]. Learning activities that incorporate obstacles, disruptions and challenges promote more profound learning, and extending knowledge often occurs in spaces of uncertainty. This kind of learning typically arises when individuals perceive that something is at stake, and it often requires them to stretch beyond their comfort zones. Vygotsky’s theory of the zone of proximal development highlights the importance of moving beyond self-mastery into areas of uncertainty and challenge [13, 14].

Reflection on experiences plays a key role in students’ learning process within SBE [15]. Simulation debriefings create opportunities for these reflections, enabling participants to give and receive feedback, verbalize their experiences and analyse their actions. However, reflection practices are most effective within a safe learning climate [16].

The establishment of psychological safety has emerged as a central focus in recent and ongoing research regarding simulation in healthcare education [1], with a growing consensus that it is a prerequisite for effective learning [4, 16–19]. While much of the literature focuses on implementation and the facilitator’s role, there is limited examination of how psychological safety is *conceptualized* and *argued for* across the SBE research landscape. This study aimed to explore how psychological safety has been conceptualized, framed and argued for in the SBE literature over the past decade.

## Methods

### Study design

An integrative review was chosen because it is an appropriate method for understanding phenomena with practical significance in a field. This method allows for the inclusion of studies employing various methodologies, offers insights into concepts from multiple perspectives and provides a comprehensive understanding, which can be particularly beneficial in the context of educational practice [20].

Whittemore and Knafl’s [20] integrative review method with five stages was performed as follows: (1) problem identification, (2) literature search, (3) data evaluation, (4) data analysis and (5) presentation.

### Search methods

The search strategy was based on an initial broad search developed by a research librarian in cooperation with the authors to ensure correct terms and combinations, according to each database. As an example, the search strategy included various entry terms relating to psychological safety, simulation and learning that were combined with Boolean operators as follows: (“psychological safety” OR “psychological safe environment”) AND (“scenario-based simulation” OR “simulation-based learning” OR “simulation training”) AND (“nursing student” OR “medical student” OR “health professional student”).

The main searches were conducted in November 2023, with no imposed date range, in the following databases: CINAHL, Medline, EMBASE, PsycINFO, EBSCO ERIC, EBSCO Education Source and Scopus. A follow-up search was conducted to identify articles published between November 2023 and the end of September 2024. Two new articles were identified but did not meet the inclusion criteria. The results from the database searches were collected using reference management software Endnote. After controlling for duplicates, the references were imported to the software screening tool Rayyan, which allowed the researchers to manually screen the abstracts independently.

### Search outcomes

After the duplicate check, the number of articles decreased from 2071 to 1493 articles, and the two authors (AM and AS) performed the screening process of 1493 abstracts blinded. Subsequently, the selected abstracts were discussed against the inclusion and exclusion criteria, as shown in Table 1. The final discussion of the two researchers resolved any disagreements, using the inclusion criteria as a guide.

**Table 1:** Inclusion and exclusion criteria

Inclusion criteria	Exclusion criteria
Simulation-based education Scenario-based learning Simulation-based learning	Virtual and gaming simulation
Health profession students	Professional health workers
Psychological safety as a premise for learning	Psychological distress Anxiety Stress Self-efficacy
Peer-reviewed articles	Books and book chapters Conference proceedings Abstract only Grey literature Reports Editorials
Published after 2013	
European and English languages	

**Fig. 1:**
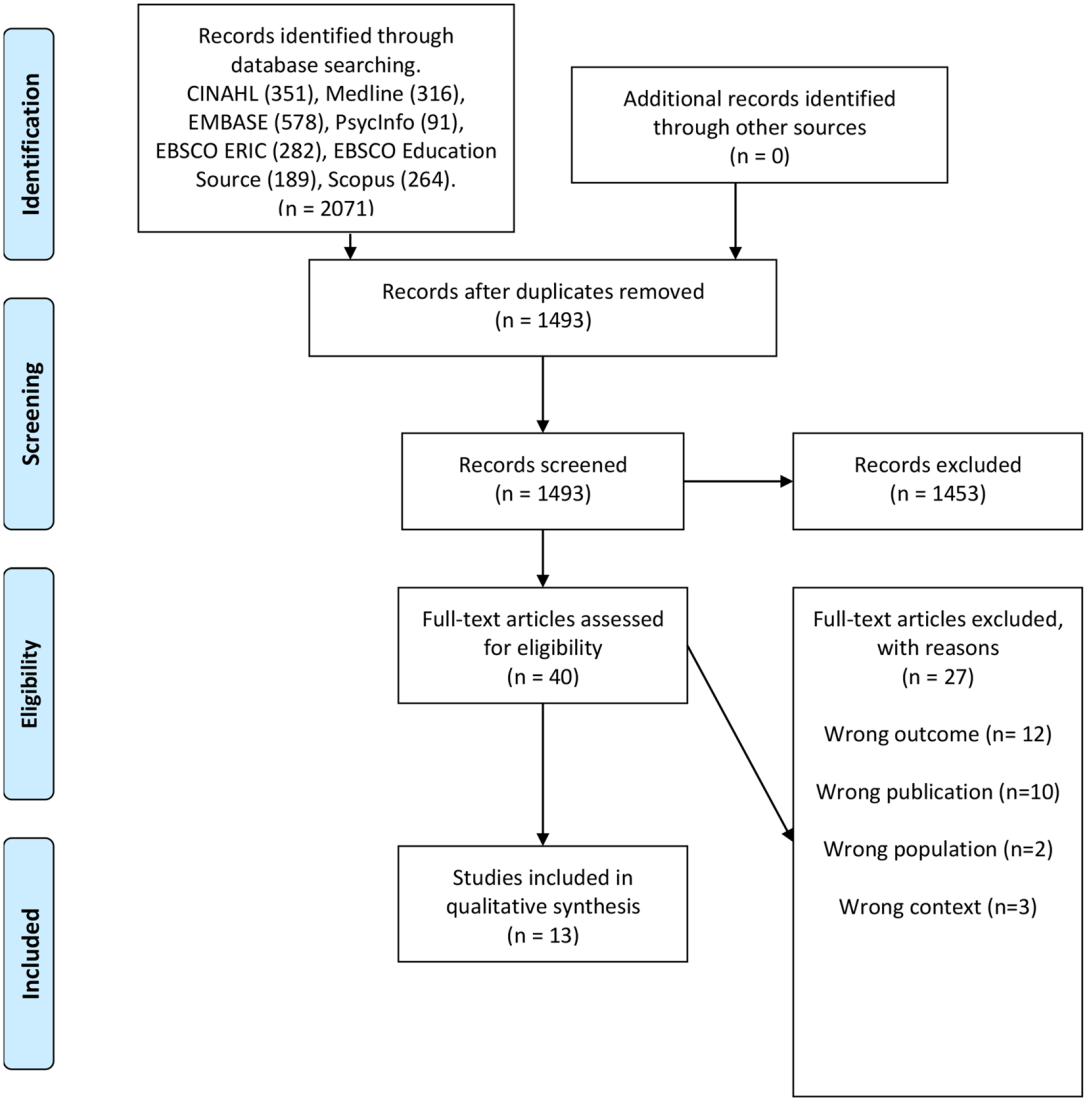
Preferred Reporting Items for Systematic Reviews and Meta-Analyses (PRISMA) flow chart illustrating the screening process

Figure 1 is a flow chart of the selection process. A total of 40 full-text articles were assessed for eligibility. Finally, 13 articles were included.

### Quality appraisal

To assess the risk of bias in the studies, the Critical Appraisal Skills Programme (CASP,2013) tool was adopted to systematically appraise the methodological quality of the included articles. Table 2 show the researchers` ratings of the 13 studies based on their presentation of design, methods, ethical quality, analyses and discussion. No studies were excluded due to substantial bias.

**Table 2:** Critical appraisal skills

	1	2	3	4	5	6	7	8	9	10
El-Hussein and Harvey (2022)	Yes	Yes	Yes	Yes	Yes	Yes	Yes	Yes	Yes	Yes
Ganley and Linnard-Palmer (2012)	Yes	Cannot tell	Cannot tell	Yes	Yes	Yes	Cannot tell	Yes	Yes	Yes
Kang and Ming (2019)	Yes	Yes	Cannot tell	No	Yes	Yes	Cannot tell	Yes	Yes	Yes
Ko and Choi (2019)	Yes	Yes	Cannot tell	Yes	Yes	Cannot tell	Yes	Cannot tell	Yes	Yes
Kostovich et al. (2020)	Yes	Yes	Cannot tell	Cannot tell	No	Yes	Yes	Yes	Yes	Yes
Madsgaard et al. (2022)	Yes	Yes	Yes	Yes	Yes	Cannot tell	Yes	Yes	Yes	Yes
Park and Kim (2021a)	Yes	Yes	Yes	Yes	Yes	No	Yes	Yes	Yes	Yes
Park and Kim (2021b)	Yes	Yes	Yes	Yes	Yes	No	Yes	Yes	Yes	Yes
Roh et al. (2018)	Yes	Yes	Yes	No	Yes	No	Yes	Yes	Yes	Yes
Roh et al. (2021)	Yes	Yes	Yes	Yes	Yes	No	Cannot tell	Yes	Yes	Yes
Spill and Gatin (2019)	Yes	Yes	Yes	Yes	Yes	No	Yes	Cannot tell	Yes	Yes
Stephen et al. (2020)	Yes	Yes	No	Yes	Cannot tell	Cannot tell	Yes	Yes	Yes	Yes
Turner et al. (2023)	Yes	Yes	Yes	Yes	Yes	Cannot tell	Cannot tell	Yes	Yes	Yes

1) Statement and aim, 2) Method appropriate, 3) Appropriate design to address aim, 4) Appropriate recruitment strategy, 5) Data collection, 6) Considers researcher/participant relationship, 7) Ethical issues, 8) Data analysis, 9) Statement of findings, 10) Valuable

### Data analysis

The analysis was conducted by the two authors, first independently and then through discussion to identify codes and themes. This analytical process focused on recognizing patterns, variations and comparisons [20, 21]. The first stage involved reading the studies, particularly the findings, to obtain a general impression of the data. Next, the data from each study were coded, incorporating both qualitative and quantitative data. Finally, the codes extracted from all the articles were compared using a matrix which involved clustering and comparing codes to identify key themes.

## Results

Table 3 describes the characteristics of the included studies (i.e. the purpose, sample, study design and findings).

**Table 3:** Characteristics of the included studies

Authors, year	Location	Purpose	Participants	Study design	Key findings
El-Hussein and Harvey (2023)	Canada	Provide a theoretical understanding of processes driving nursing students’ actions during the simulation	Nursing students *N* = 32	Qualitative	Highlights the value of a psychologically safe learning environment in which collaborative reasoning can occur without fear of embarrassment or reprisal. Safety was supported through reprieving and empowering practices, with prebriefing and the facilitator’s role emerging as critical to shaping the students’ experiences. Tailored support and consolidation of knowledge were essential to contextualizing psychological safety in simulation.
Ganley and Linnard-Palmer (2012)	USA	Report students’ ideal and actual experiences in high-fidelity simulation	Nursing students *N* = 101 Faculty *N* = 24	Quantitative	The students reported experiencing less academic safety, comfort and greater anxiety than the faculty anticipated. While the students described a safe learning environment as one free from ridicule, embarrassment or paralyzing fear of failure, the faculty tended to define safety more holistically as a setting where students are supported and challenged without being threatened.
Kang and Ming (2019)	South Korea	Understand nursing students’ perceptions of psychological safety in simulation	Nursing students *N* = 15	Qualitative	The students expressed reluctance to participate in simulation due to feeling unprepared. The perception of being evaluated during the simulation intensified their fear and anxiety, negatively impacting their learning experiences.
Ko and Choi (2020)	South Korea	Explore nursing students’ stress in simulation and experiences of psychological safety	Nursing students *N* = 23	Qualitative	Stress was acknowledged as a potential learning catalyst.
Kostovich et al. (2020)	USA	Explore faculty’s perceptions of psychological safety in simulation	Faculty *N* = 37	Qualitative	Facilitators promote a safe learning environment in SBE by forming small groups, providing clear prebriefing, ensuring emotional support, prioritizing confidentiality and viewing mistakes as valuable learning opportunities.
Madsgaard et al. (2022)	Norway	Investigate how facilitators approach students’ emotions in SBE	Faculty *N* = 9	Qualitative	Facilitators balance psychological safety with emotional engagement to optimize learning.
Park and Kim (2021a)	South Korea	Examine students’ experiences of psychological safety and risks in simulation education	Nursing students *N* = 20	Qualitative	The students’ anxiety was linked to uncertainty, exposure, lack of support and taking interpersonal risks.
Park and Kim (2021b)	South Korea	Measure psychological safety in simulation-based learning settings using high-fidelity manikins	Nursing students *N* = 242	Quantitative	A scale measuring psychological safety in simulation was developed and validated, focusing on the students’ experiences with uncertainty, disrespect, risks associated with team dynamics and exposure. The scale was found to be both reliable and valid, offering valuable support for nursing educators aiming to foster psychological safety in SBE.
Roh et al. (2018)	South Korea	Examine effects of prebriefing on team psychological safety and performance	Nursing students *N* = 281	Quantitative	Prebriefing activities with concept mapping for patient care resulted in higher levels of team psychological safety and improved cardiopulmonary resuscitation performance compared to those activities without concept mapping.
Roh et al. (2021)	South Korea	Explore psychological safety mediating relationship between simulation design features and learning outcomes	Nursing students *N* = 194	Quantitative	Perceptions of psychological safety positively correlate with learning outcomes, with simulation design features being a significant predictor.
Spill and Gatin (2019)	France	Investigate tutors’ strategies for establishing psychological safety during emergency simulation	Faculty *N* = 6	Qualitative	Reassurance and care without judgement were emphasized, especially for students feeling terrified or scared. Strategies included thorough briefing, humanizing themselves, using humour, changing the context and addressing students by their first names.
Stephen et al. (2020)	Canada	Explore students’ perceptions of psychological safety across all phases of a simulation learning experience	Nursing students *N* = 86	Qualitative	A supportive, realistic, and judgement-free environment was emphasized, with small groups, clear expectations and positive conversations.
Turner et al. (2023)	Canada	Understand perspectives of students and facilitators on psychological safety in simulation	Nursing students *N* = 7 Faculty *N* = 4	Qualitative	Discomfort and judgement based on past performance led to reluctance to take risks or participate. Effective communication was free from critical, judgemental or negative language, with facilitators emphasizing trust-building, confidentiality and creating a ‘safe bubble’.

### Descriptions of studies

A total of 13 studies were included in the analysis. The studies originated from 5 countries: 6 from South Korea, 3 from Canada, 2 from the USA and 1 each from France and Norway. Among the studies, 9 used qualitative methods and 4 used quantitative methods. The data collection methods used included 5 studies that used semi-structured or in-depth interviews, 6 that conducted surveys, 2 that held focus group interviews and 1 that used video recorded analysis. A total of 12 studies had nursing student participants, while 5 studies had faculty participants, either alone or in combination with students.

Psychological safety was the core theoretical element in all 13 studies. Edmondson’s definition of psychological safety was referred to in 7 studies as the theoretical framework, while 2 studies used the term ‘academic safety’. A theoretical perspective underpinned only 1 study, which referred to Kolb’s experiential learning cycle. The simulation designs used were the National League for Nursing/Jeffries simulation framework in 5 studies, the International Nursing Association for Clinical Simulation and Learning simulation design in 3 studies, and the Society for Simulation in Healthcare’s framework in 2 studies.

### Psychological safety in simulation-based education

All 13 studies emphasized the importance of creating a psychologically safe learning environment in response to students’ reports of stress and anxiety during SBE. The analysis highlighted two key themes that emphasize the importance of psychological safety for learning during SBE.

### Theme 1: psychological safety in an unsafe learning environment

Faculty aimed to establish a psychologically safe learning environment in response to reports detailing students’ experiences of stress, fear, discomfort, anxiety, nervousness and feelings of unsafety [22–32]. Several factors contributed to students’ sense of unsafety during SBE. They reported uncertainty about what to expect, unfamiliarity with the learning situation, discomfort with being videotaped and feeling rushed. Additionally, they mentioned experiencing unpreparedness, due to lack of necessary knowledge and practical skills. Another factor was students anxiety about making mistakes in front of others, discomfort with being observed, pressure to perform effectively as a team and fear of assessment which may be significant sources of stress [23, 24, 28, 33].

Facilitators employed various strategies to address students’ feelings of unsafety. They acknowledged that identifying anxious students was challenging, as these students often did not verbalize their fears. Instead, facilitators recognized students’ anxiety by observing the students’ body language, verbal feedback and behaviour [27, 31]. Students indicated that effective communication and an experience of safety were fostered when facilitators used language that was not overly critical, judgemental or negative [34]. One study highlighted the need for SBE to strive to “avoid any uncomfortable feelings or hurtful situations” [28], and another study emphasized that facilitators should endeavour to run simulations in the absence of students’ anxiety and avoid exposing or evaluating students in a way that causes discomfort [29].

Facilitators implemented strategies to create a safe atmosphere by sharing their own simulation experiences, offering reassurance, demonstrating care and maintaining a non-judgemental approach [31]. They also adjusted the difficulty level and assigned roles to students who were comfortable being in the spotlight [27, 31]. Interestingly, students reported experiencing more discomfort and anxiety during SBE than the faculty anticipated [23]. Several studies recommend strategies for creating a psychologically safe environment during simulations. Facilitators are encouraged to establish the simulation as a safe place during the prebriefing and briefing stages of the simulation [22, 26, 30, 31, 35]. Key practices include setting clear expectations, outlining what will occur during simulation, defining roles, ensuring confidentiality and fostering a non-threatening atmosphere. Furthermore, tailoring support, consolidating knowledge, promoting respectful interactions and organising small groups are all critical elements for maintaining a safe learning environment [22, 27, 34].

### Theme 2: psychological safety as a prerequisite for learning

Ensuring that SBE is a safe arena for learning was underscored as crucial because an insecure environment negatively impacts students’ ability to fully participate in discussions and take risks. When students did not feel psychologically safe during simulation, they were found to be less likely to speak up or actively engage [34]. Psychological safety contributed to reducing students’ stress levels, which were found to be essential for improving cognitive function and critical thinking [22]. The correlation between psychological safety and positive learning outcomes was documented by the fact that students who reported feeling safe in SBE environments achieved better learning results [30].

Four studies emphasized that establishing a psychologically safe learning environment was crucial for challenging students to catalyze learning [26–28, 33]. Facilitators reported that SBE provided opportunities to create learning situations with uncertain outcomes. They emphasized the importance of establishing a psychologically safe environment in which students feel confident enough to risk failure. The value of students encountering failure or making mistakes during SBE was highlighted as a factor in enhancing learning [26, 27]. Students reported that failure became a valuable learning experience when they were not embarrassed but instead supported by their peers and free from the fear of failure [23]. Facilitators were found to carefully balance challenges and safety during SBE to optimize students’ learning [23, 27], and adjusted the complexity and demands of the simulation as needed when the students appeared insecure or stressed. This delicate balance involves pushing students to confront challenges without making them feel threatened or overwhelmed [23].

Students reported that they wanted to be challenged beyond their comfort zone and experience healthy anxiety; they appreciated it when their mistakes were treated as learning opportunities rather than sources of ridicule [23]. Learners who seek challenges in SBE may experience increased confidence as well as enhanced learning when their anxiety is positively channelled. One study emphasized that an optimal level of stress could sharpen students’ focus, improve their learning outcomes and enable them to gain clinical experience [33]. Another study highlighted the strategy of emotional eliciting to challenge students and improve their learning outcomes [25].

## Discussion

Our analysis of the 13 studies provides insights into the importance of psychological safety in ensuring a supportive learning climate during SBE. The findings underscore the recognition of the challenges nursing students face during SBE and the necessity of creating a safe learning environment.

### Importance of psychological safety in SBE

Most studies in this review considering psychological safety in SBE embrace the protective and safety aspects of the concept and findings highlight the critical role of psychological safety in ensuring a supportive learning climate during SBE. Students’ feelings of exposure guide facilitators to prioritize the safety aspect in the leaning process when implementing SBE. The findings indicate that faculty consider psychological safety to be crucial in SBE, especially since students experience SBE as an anxiety-provoking setting in which they feel exposed [26–28, 33]. From the student perspective analysis highlights that students often perceive SBE as a learning environment in which they are highly exposed, leading to feelings of discomfort [22–32].

### Challenges within the learning environment

While psychological safety is essential for fostering learning, students also emphasize the need to be challenged, experience comprehensive anxiety and learn from their mistakes [23]. The findings suggest that while simulations can be uncomfortable and psychological safety is crucial for learning, effective learning requires challenging students beyond their comfort zones [26, 27, 33]. The goal of simulations is not to create a risk-free and comfortable environment, as this may diminish the overall learning experience [10]. An excessive focus on safety can lead to complacency, whereby students avoid taking the risks necessary for profound learning experiences [4]. SBE, which is a socio-cultural learning environment, requires a certain level of security to foster active participation, social interaction and engagement. However, this security should not come at the expense of challenging students and pushing them beyond their comfort zones [10, 14]. Excessive safety without corresponding demands and expectations can hinder the learning process.

The findings of this study emphasize the necessity of presenting challenges to students in a psychological safe learning environment [26–28, 33], a concept supported in pedagogical literature. A socio-cultural learning perspective supports the notion that psychological safety must be paired with challenges and expectations to be truly effective [10, 11]. An overly safe environment in SBE can reduce students’ motivation and drive for excellence, leading to mediocrity rather than mastery. Educational growth frequently occurs outside one’s comfort zone; by avoiding the provision of challenging experiences, educators may inadvertently limit students’ preparation for the complexities of practice beyond the classroom [4, 11, 36]. Moreover, creating challenging learning environments is important formeaningful experiences and reflective learning [11]. When students encounter challenges and dilemmas, they may experience dissonance between previous knowledge and unfamiliar knowledge, resulting in a gap between their prior understanding and the frames of reference they bring to the situation [11]. This dissonance serves as a catalyst for learning, driving students to actively seek knowledge and explore solutions to the dilemmas they face [13].

While there is a substantial focus in the literature on creating safe learning environments, it is important to note that theory on psychological safe learning environment involves more than simply being agreeable; it also encompasses the challenges necessary for growth [4]. Therefore, creating psychologically safe simulations involves providing a supportive yet challenging atmosphere [4, 37]. Reflection on experience is of utmost significance for learning during SBE [15]. The reflection process becomes more profound when students contemplate experiences that involve a consequential element and delve into these experiences rather than simply reflecting on their actions [9]. SBE provides an environment in which students can learn without fear of causing harm to actual patients [1]. This allows for the creation and implementation of scenarios that simulate experiences with consequences, leading to deeper reflection rather than mere surface-level reflection. This allows for the creation and implementation of scenarios that simulate consequential experiences that may be difficult to encounter during actual patient practice, fostering a more profound reflective practice.

### Balancing safety and challenges

Our findings indicate that psychological safety in SBE is not solely about ensuring that students feel secure or avoiding students’ discomfort. It also involves facilitators creating an environment that balances support with appropriate challenges [26–28, 33]. These challenges should be tailored to align with students’ resilience, education level, existing knowledge and the complexity of the simulated situation. Vygotsky’s theory of the zone of proximal development [14] emphasizes that learning is optimized when challenges are perceived to be moderate and achievable.

Ganley and Linnerard-Palmer [23] highlight that an academically safe learning environment encompasses not only physical safety but also emotional, psychological and social dimensions. By observing and understanding students’ experiences, facilitators can adjust the difficulty level to align with each student’s sense of security, thereby promoting learning without overwhelming them. Facilitating learning in simulation requires facilitators to master balancing between ensuring safety while setting demands and creating challenges [27]. Mastering this balancing act is critical for facilitators and hinges on facilitators’ pedagogical expertise and ability to adapt in response to students’ reactions in simulation [37].

Additionally, simulation is deeply influenced by the surrounding learning culture, which is built on students’ understanding of the educational rationale and their confidence in facilitators’ intentions. A positive learning culture fosters confidence in the pedagogical choices made and reinforces the belief that facilitators are committed to students’ success [37]. It is equally important for students to comprehend and accept the rationale behind the expectations and challenges they will encounter. Therefore, psychological safety should permeate the learning culture and not be exclusively related to SBE [38]. Establishing a psychologically safe learning culture should extend beyond specific learning situations and be ingrained in education. Resilience is essential for nurses to manage the demands and pressures of clinical practice [39]. Psychological safety can support the development of resilience in students, preparing them to withstand challenges and handle stress. Rather than simply adjusting learning situations to avoid discomfort, students can be equipped with the skills and mindset needed to navigate and learn from adversity [11].

Moreover, the implications of the review findings show that the potential of SBE as a context for fostering opportunities to challenge students should be further explored. Given its structured nature and controlled learning environment, simulation provides a unique opportunity to introduce dilemmas and meaningful patient scenarios that push students beyond their comfort zones. While ensuring comfort and reducing distress are important, recommended practices should also emphasize the creation of challenging simulation situations within a safe environment. Although psychological safety is fundamental to learning in complex, socially interactive and uncertain simulation contexts, its implementation could be more refined, recognizing that it is not merely about eliminating all discomfort.

### Methodological considerations

One notable strength of this study is the involvement of two researchers with experience in simulation and facilitation during the analysis. This expertise ensures quality, critical evaluations of the included studies and coherence in the data interpretation. To make sure all studies related to psychological safety in SBE involved students, we conducted a comprehensive search. Each researcher independently included or excluded the 1493 studies.

The involvement of a librarian ensured that quality checks were made on the search strategies, which further enhances the study’s rigour and reliability.

One limitation is that we included only studies published after 2013. However, this is because, SBE, as we know it today as an education method, did not emerge until the early 2000s. Furthermore, the concept of psychological safety is quite new in the SBE context.

In terms of limitations, it is challenging to compare concepts such as psychological safety across various educational systems, facilitation styles and between different cultures. The diverse facilitation styles used by different facilitators in the 13 studies included in this research may have influenced students’ experiences of psychological safety during SBE. This is a variable beyond our control and could impact the overall perception of psychological safety among students. Furthermore, individual differences among students, such as their ability to manage stress, prior knowledge, varying competencies and cultural differences can also affect their perception of safety. In studies performed in different cultural contexts. it is important to recognize that the concept of psychological safety can vary depending on the cultural context in which it is experienced.

## Conclusion

In summary, this review underscores the relevance and necessity of psychological safety in SBE, highlighting that it is a key premise for learning. Psychological safety enables students to engage actively and reflect during SBE, but it must be balanced with adequate challenges. While ensuring that students feel supported is essential, it is equally important to introduce challenges and risks that promote growth and development. Thus, educators must consider whether their efforts to create an exclusively psychologically safe environment might limit students’ potential for deeper learning and critical thinking.

Moving forward, the concept of psychological safety in SBE should be guided by pedagogical frameworks and learning theories, acknowledging that safe yet challenging simulation environments are key to fostering meaningful educational outcomes.

## Data Availability

All the data are available from the corresponding author up on a reasonable request.
